# M2 Macrophages Serve as Critical Executor of Innate Immunity in Chronic Allograft Rejection

**DOI:** 10.3389/fimmu.2021.648539

**Published:** 2021-03-17

**Authors:** Hanwen Zhang, Zhuonan Li, Wei Li

**Affiliations:** ^1^Department of Hepatobiliary-Pancreatic Surgery, China-Japan Union Hospital of Jilin University, Changchun, China; ^2^Plastic Surgery, China-Japan Union Hospital of Jilin University, Changchun, China

**Keywords:** solid organ transplantation, chronic allograft rejection, macrophage, IRI, inflammation, ECM, fibrosis, vasculopathy

## Abstract

Allograft functional failure due to acute or chronic rejection has long been a major concern in the area of solid organ transplantation for decades. As critical component of innate immune system, the macrophages are unlikely to be exclusive for driving acute or chronic sterile inflammation against allografts. Traditionally, macrophages are classified into two types, M1 and M2 like macrophages, based on their functions. M1 macrophages are involved in acute rejection for triggering sterile inflammation thus lead to tissue damage and poor allograft survival, while M2 macrophages represent contradictory features, playing pivotal roles in both anti-inflammation and development of graft fibrosis and resulting in chronic rejection. Macrophages also contribute to allograft vasculopathy, but the phenotypes remain to be identified. Moreover, increasing evidences are challenging traditional identification and classification of macrophage in various diseases. Better understanding the role of macrophage in chronic rejection is fundamental to developing innovative strategies for preventing late graft loss. In this review, we will update the recent progress in our understanding of diversity of macrophage-dominated innate immune response, and reveal the roles of M2 macrophages in chronic allograft rejection as well.

## Introduction

Organ transplantation appears to be the best treatment method for patients who suffered from the end-stage diseases. However, chronic allograft rejection following transplantation remains to be a major challenge to long term allograft survival and functions. Organ allografts are susceptible to ischemia reperfusion injury (IRI) and recipient alloimmunity, suffering from acute and chronic rejection inevitably (1). Much attention has once focused on acute rejection, but nowaday it is no longer the common cause of late graft loss due to the application of systemic immunosuppressants (2). Increasing evidences are focusing on the profound meaning of innate immune cells, such as NK cells, macrophages, and neutrophils, in the late phase change of allografts.

Diversity of innate immune cells is drawing much attention. Neutrophils have been characterized as a major component in driving inflammation for a long time. They are known to be recruited into stressed solid organ and contribute to tissue injury, while recent data have demonstrated their contradictory function in tissue repair (3, 4). Identically, distinctive functional features between two different populations of macrophages are considered as a hot spot to study in varies diseases models. Despite the fact that macrophages are recognized as a key participant in triggering both graft acute inflammatory damage and inflammation resolution in organ transplantation (5). The role of macrophage-mediated late phase graft rejection and corresponding therapies to mitigate allograft dysfunction or loss still remain largely unknown.

Monocyte-derived macrophages infiltrate into allografts after transplantation and then mainly differentiate into two phenotypes: pro-inflammatory M1-like macrophages and anti-inflammatory M2-like type (6), thus represent opposite functions during acute phase in organ transplant. However, M2-like macrophages also show potentials for enabling fibrosis, resulting in poor long-term graft survival. Moreover, macrophages are reported to be related with graft vasculopathy (7), but the data on their heterogeneity are limited. Tissue-resident macrophages are displaying differential features compared with their infiltrating counterpart. Although it has been proved that the replenishment of local macrophage niche in organ under unsteady state, increasing data have shown the differential characteristics and features between embryonic and monocyte-derived populations (8). Diverse macrophages dynamically represent various functions in local immune environment in response to stimuli, stress and tissue injury, while a great deal of work is yet to be done in the area of chronic allograft rejection. An improved understanding of macrophage related cellular immune events which trigger the late phase rejection and ultimately responsible for allograft dysfunction and loss, is fundamental for the development of innovative strategies for the treatment of organ allograft recipients. In this review, we will update the recent progresse of diversity of macrophage dominated innate immune responses and reveal the roles of M2 macrophages in chronic allograft rejection as well.

## Identification of Macrophages

### Distinct Origins and Functions of Macrophages

As we all know so far, both innate and adaptive immune responses are involved in chronic allograft rejection. The key effective innate immune cells, macrophages, play important roles in variety of physiologic and pathologic processes, for instance, host defense, acute and chronic inflammation and tissue repair (9). Macrophages are derived from monocytes in the circulation and then represent resident features in lymphoid and non-lymphoid tissues, including solid organs, involved in tissue homeostasis (10). They were characterized by plasticity and diverse functions. The activated macrophages polarize and exhibit classical M1-like or alternative M2-like phenotypes. M1 macrophages tend to produce reactive oxygen species (ROS), nitric oxide (NO) and pro-inflammatory cytokines such as IL-6, IL-12, and TNF-α that play crucial roles in defense against dead cells, microbial infection and cancer. On the contrary, M2 macrophages tend to express scavenging receptors and produce various anti-inflammatory mediators including IL-10 and TGF-β to promote the inflammation resolution, tissue repair, wound healing and fibrosis (11).

**Figure 1:**
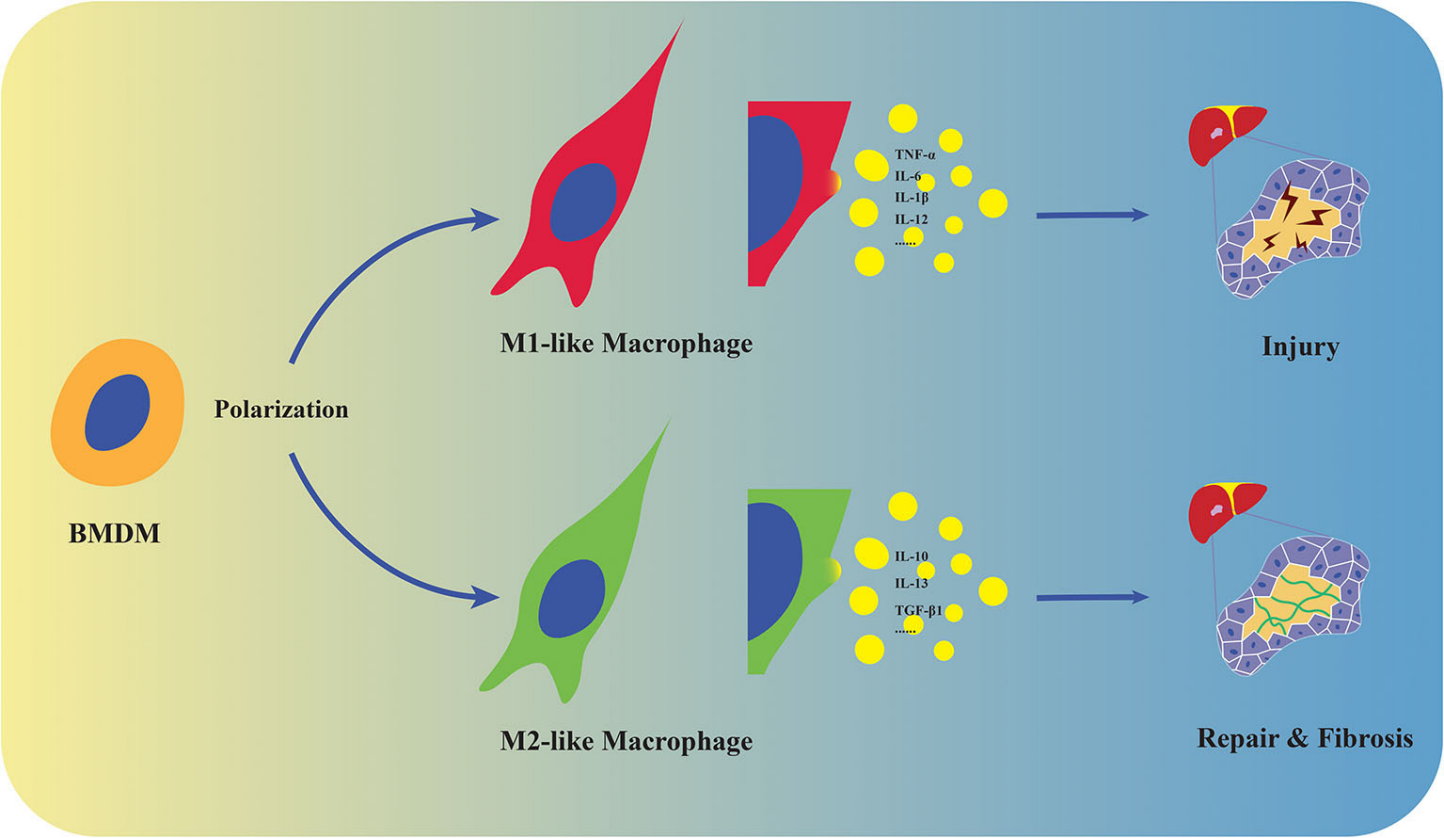
Polarization and distinct functions of macrophages. Macrophages derive from monocytes in the circulation and polarize into M1/M2-like phenotypes. M1-like macrophages promote tissue injury by producing pro-inflammatory cytokines including TNF-α, IL-6, IL-1β, and IL-12, while M2 represent tissue repair and fibrosis by expressing anti-inflammatory and fibrogenic mediators such as IL-10, IL-13, and TGF-β1 in stressed liver.

Traditionally, the polarization of macrophage may be defined by M1/M2 markers including Nitric Oxide Synthases (NOSs), arginase family (Arg1/2), CD206 and CD163. In spite of that, macrophages are likely to represent distinctive polarization and differentiation under various circumstances in complex organism. Understanding dynamic reprogramming landscapes of macrophage is important for exploiting the mechanisms of transplant immune responses (Figure 1). Of note, a unique macrophage subset driven by IL-23 has been recently discussed. IL-23, a heterodimeric cytokine essential for expression of IL-17, shares p40 subunit with IL-12 that induces the production of IFN-γ (12). It was reported that IL-23 involved in IL-17 and IFN-γ related tissue inflammatory response by innate lymphoid cells, including macrophages (13, 14). Treated with recombinant IL-23, unpolarized mouse peritoneal macrophages represent significantly increased production of IL-17A and IFN-γ via STAT3-RORγ T pathways, but not M1/M2 related cytokines, and neither polarized M1 nor M2-like macrophages can convert to such an IL-17/IFN-γ high-producing subset: M(IL-23) (15). Thus, M(IL-23) is likely to be defined as a new macrophage subpopulation and is worth discussing since alloreactive production of IL-17/IFN-γ is closely related to allograft rejection (16).

### Dynamic Change Among Resident Macrophage Niche in Allograft Rejection

Tissue resident macrophages are crucial participants in organ homeostasis and are likely to exhibit a M2-like phenotype (17). However, M2-like macrophages could also display pro-fibrogenic feature. In other word, tissue-resident macrophages are double-edged swords that have potential to either alleviate IRI and inflammation, or result in fibrotic rejection. Recently, many studies are focusing on postnatal development of tissue resident macrophages and their functions in metabolism, immune activation and regulation. Unlike primitive tissue-resident macrophages such as Kupffer cells (KCs) in the liver, microglia in brain and renal resident macrophages that arise from yolk sac (18–20), macrophages originate from bone marrow-derived monocytes and then reside in solid organs, serving as executors of innate immunity. Identical to liver parenchyma cells, non-parenchyma cells (NPCs) in the liver suffer from hypoxia, leading to ischemia-induced necrotic activation (21). Although it has been widely acknowledged that immigrant macrophages and tissue-resident macrophages display distinct features and properties in some aspects, niche-specific reprogramming of recruited macrophages with the help of “KC-enhancers” expressed by liver sinusoidal endothelial cells and hepatocytes have been recently discovered on the basis of “resident like” marker V-set immunoglobulin-domain-containing 4 (VSIG4) (22, 23). Tissue resident macrophages are susceptible to endo-/exogenous stimulation and contribute to necrotic or apoptotic depletion. Tremendous findings have demonstrated that the potentials on the transformation of the immigrant populations into the resident are not only compensating impaired resident macrophage pool, but also representing resident-like features and functions that “newcome” macrophages lack in response to stress including surgery-induced IRI, infection and chronic inflammation (17, 22, 24). Despite the fact that limited works have focused on macrophage reprogramming in organ transplantation, progress pertaining to the aforementioned factors suggest the innovative possibility in alleviating macrophage-mediated chronic allograft rejection.

## Macrophages, Inflammation and Chronic Allograft Rejection

### Innate Immunity Dominated IRI in Transplantation

IRI which triggered by activation of innate immune receptors, not only play a key role in graft failure at the early stage, but also result in chronic rejection at the late stage of transplantation (25). This inevitable complication initiates tissue damage by promoting release of danger-associated molecular pattern (DAMP) such as high mobility group box-1 (HMGB1) which derived from necrotic or stressed cells to activate sentinel pattern recognition receptor Toll-like receptor 4 (TLR4), thus mediating sterile inflammation (26, 27). As the major innate immune cells responding to DAMPs, monocyte-derived macrophage are activated and infiltrate into IR-stressed organs, which is closely associated with inflammation and tissue injury. However, the functions of macrophage are different in their population and may represent distinct roles in response to IRI stress. It has been revealed based on the biological phenotype of macrophage that M1-like macrophages are responsible for inducing neutrophil-mediated tissue injury, while M2-like phenotype monocyte-derived macrophages and kupffer cells are likely to represent anti-inflammatory and wound-healing features during liver IRI (28, 29).

### Macrophages in Inflammation and Allograft Rejection

Compared with monocyte-derived macrophages originated from bone-marrow progenitor cells and take time to be recruited from circulation, tissue-resident macrophages represent rapid response. They sense neighboring cells death and react to DAMPs as soon as they are released by dead organ parenchymal cells, directly function on cellular debris degradation and cytokines secretion against injury (25). More notably, they are stimulated and activated during ischemic stage before the recruitment of monocyte-derived macrophages into stressed organ. Thus, diverse macrophage subsets and complex innate immune activation demonstrate a complicated scenario, converting immunologically quiescent milieu into an inflammatory activation vs. resolution condition and resulting in tissue damage, injury repair and fibrosis.

M1-like macrophage-dominated innate immune response is one of the major sources of acute neutrophil-mediated inflammation in transplant recipients. Indeed, study using transgenic cre-inducible diphtheria toxin receptor (DTR) system that specifically targets CD11b^+^ (a surface marker expressed on macrophages) cells *in vivo* to deplete infiltrating macrophages demonstrated the alleviative effect on liver IRI in mice (30, 31). At early stage of post-transplant, overwhelming innate immune response dominates acute rejection, while T-cell mediated adaptive allograft rejection has not yet been established. M1-like macrophages are inclined to produce pro-inflammatory cytokines TNF-α, IL-6, and IL-1β, mediating injury. As pro-/anti- inflammatory activation and tissue repair co-exist, implants undergo dynamic change. Failing to resolve acute inflammation may result in chronic inflammation, attributing to establishment of allograft inflammatory fibrosis and gradual deterioration in functions (32).

## Macrophages, Fibrosis, and Chronic Allograft Rejection

### Fibrosis and Solid Organ Diseases

Upon injuries or diseases, there is an impressive repertoire of machinery in place for self-preservation and healing. One of the most important protective mechanisms for wound healing and tissue regeneration is the formation of extracellular matrix (ECM). As a highly dynamic construction, ECM undergoes an independent remodeling process, contributes to restructuring of tissue architecture in regarding to maintenance of stable organ structure and function (33, 34). ECM unbalance caused by endo-/exogenous risk factors is likely to result in disorganization or dysfunction. Fibroblasts, one of the most abundant cell types are widely distributed in the connective tissue throughout the body. They are acknowledged to compose the basic framework for tissues and organs by producing, maintaining and reabsorption of ECM, but act as circumstantial effectors that unfrequently function expression and remodeling of ECM (35). Under homeostasis, fibroblasts remain relatively quiescent, while in response to stress and stimuli, they adapt to microenvironment and are activated in coordination with inflammation related cytokines, especially TGF-β1, by differentiating into their apex stage, myofibroblasts (36). In response to tissue injury or chronic inflammation, myofibroblasts with a terminally differentiated phenotype not only extensively produce ECM, but also inherit a contractile apparatus in order to manipulate ECM fibers physically that fill the wounds and attribute to acute or chronic inflammatory damage (37).

Fibrosis is a shared and central part of numerous pathologies including autoimmunity, metabolic disorders and graft rejection (38–40). Tissue fibrosis is a dangerous condition and pathological manifestations characterized by an excessive accumulation of ECM in response to acute/chronic injuries. In addition, interstitial fibrosis and tubular atrophy (IF/TA) of kidney cortex is best known as its paramount importance in development of poorer renal function and outcome in chronic kidney disease (CKD) and kidney transplantation (41, 42). In spite of the fact that emphasizing the predominated effects of adaptive antibody-mediated rejection (AMR) in loss of implants, innate-immune-dominated fibrotic rejection triggered by macrophages should not be neglected (43). Diverse populations of macrophage represent functionally distinction during early and late phases post-transplantation. When it comes to fibrosis, they controversially drive spectrum of phenotypes that display both pro- and anti-fibrotic functions.

### M2-like Macrophages Are Responsible for Driving Fibrogenesis

**Figure 2:**
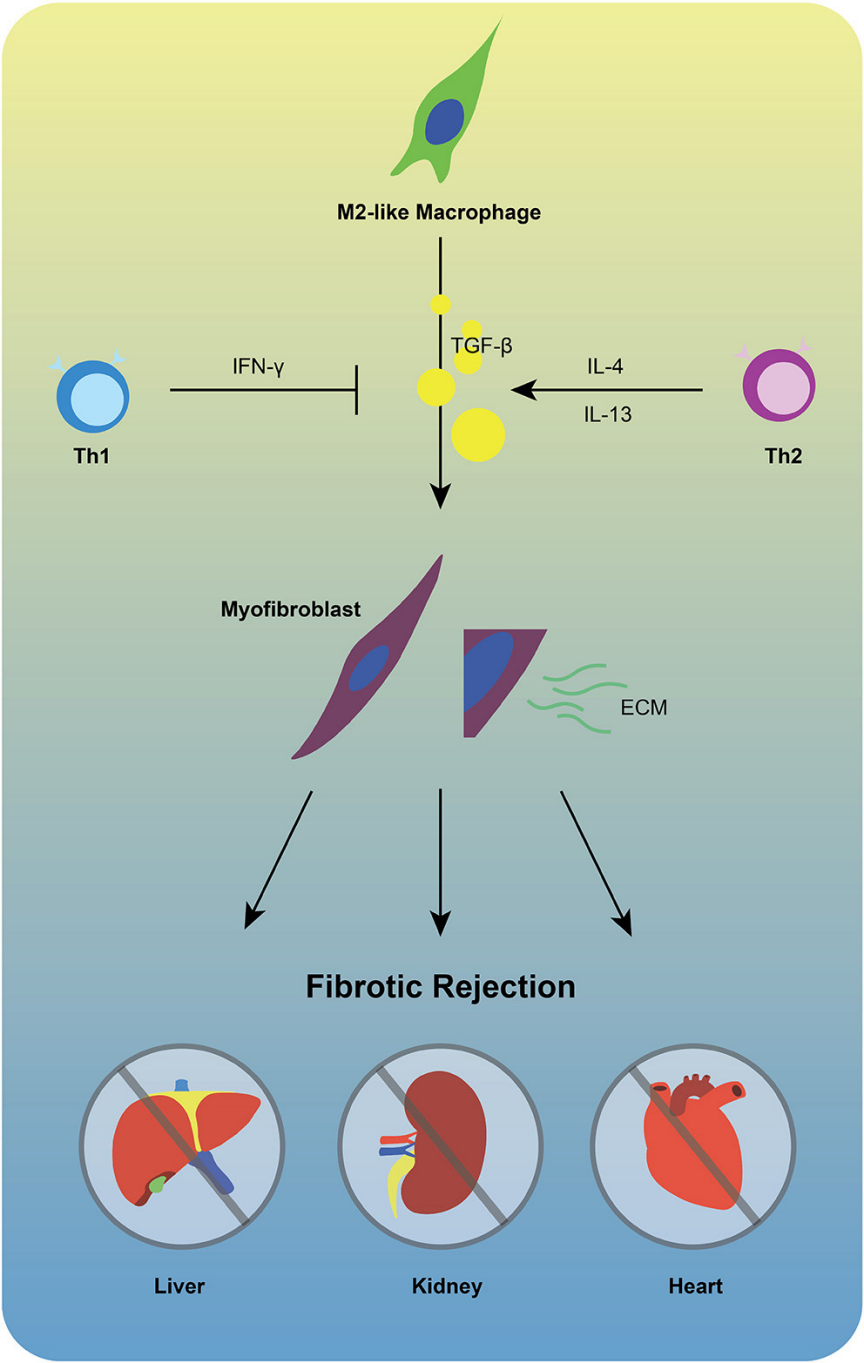
M2-like macrophages are unlikely to be exclusive for fibrotic rejection. TGF-β1 produced by M2-like macrophages facilitates differentiation of fibroblasts into myofibroblasts that extensively produce ECM, promoting chronic fibrotic allograft rejection. During this process, Th1 cells may inhibit the activation of TGF-β by producing IFN-γ, while Th2 cells represent opposite feature via secretion of IL-4/IL-13.

In the process of chronic rejection, imbalance between pro- and anti-fibrosis play an important role. Wound healing M2-like macrophages not only play critical roles in anti-inflammation in the very early stage of IRI, but also regulate tissue inflammation resolution and promote fibrotic processes via producing TGF-β1 and VEGF (44). Infiltrating macrophages differentiate into M2-like feature is likely to produce IL-10, which is crucial for suppressing pro-inflammatory gene programs. A clinical study obtained renal allograft biopsies from 12 month post-transplant, demonstrating the strong relationship between area of fibrosis and numbers of CD206^+^ M2-like macrophages (45). Smad-3 is an intracellular molecule facilitates transmit chemical signals from plasma membrane to the nucleus, operating downstream of growth/differentiation factors including TGF-β, activin, and myostatin (46). In the development of chronic kidney allograft rejection, Smad-3-dependent macrophage-myofibroblast transition (MMT) process, which occurs predominantly within M2-like macrophages, resulting in renal interstitial fibrosis (47, 48). Moreover, a novel adenosine triphosphate (ATP)-gated ion channel protein P2X7, initially reported to function in fast synaptic transmission and lysis of antigen-presenting cells (APCs), is recently found to play important roles in neutrophil adhesion, contributes to acute/chronic inflammation and M2-like macrophages-mediated chronic heart fibrotic rejection (33, 49–51). During the progression of fibrotic rejection, macrophages derived TGF-β undoubtedly play a pivotal role in differentiation of fibroblasts into myofibroblasts, but whether and how adaptive immune system affect this process remain worth studying. T helper type 1 cells (Th1 cells), defined by their secretion of IFN-γ, have potential to disrupt TGF-β/Smad3 signaling pathway-mediated fibrosis (52, 53). On the contrary, T helper type 2 cells (Th2 cells) is likely to promote the TGF-βmediated fibrosis via the production of IL-4 and IL-13 (54). Thus, further investigating the interaction between innate and adaptive immune systems in fibrotic rejection might be of great benefits (Figure 2).

## Macrophages, Vasculopathy and Chronic Allograft Rejection

### Vasculopathy Is a Hallmark of Chronic Allograft Rejection

In spite of progresses in immunosuppressive therapy, long-term success of allogeneic organ transplantation is limited by the establishment of allograft vasculopathy. Vasculopathy is another dangerous risk factor and hallmark of chronic allograft rejection triggered by multifactorial immunologic events, characterized by endothelial injury and dysfunction, myointimal hyperplasia and extracellular matrix synthesis (55, 56). Transplant vasculopathy inevitably occurs in ~90% of allografts, developing severe intimal hyperplastic lesions that eventually result in luminal flow restriction and graft failure during long-term follow-up (57). In line with this scenario, cellular and antibody-mediated rejection processes and anti-HLA antibodies against implant play crucial roles in mediating inflammation that drive the development of vasculopathy-induced chronic allograft failure (58). Transplant vasculopathy has long been considered to consequent to chronic allograft rejection, especially kidney and heart (59, 60). Nearly one-third of patients developed cardiac allograft vasculopathy (CAV) by 5 years post-transplant, leading to 12.5% CAV-induced death rate (61). Moreover, it is demonstrated that the renal vasculopathy characterized by perivascular leukocyte infiltration and neointimal hyperplasia is unlikely to be exclusive for affecting the intrarenal blood vessels (62).

### Macrophages Are Unlikely to Be Exclusive for Vasculopathy-Related Rejection

It has been reported by Croker et al. (63) that infiltrating macrophage-based post-transplant inflammation played a crucial role in chronic allograft nephropathy and dysfunction. Transplant vasculopathy is referred to as an accelerated pathologically fibroproliferative process characterized by smooth muscle proliferation and lipid deposition-induced circumferential intimal thickening (61, 64). Studies define macrophage as predominant cell type in the intimal of renal allograft arteries and demonstrate their unfavorably effects on graft survival in both acute and chronic rejection (65, 66). Depleting or inhibiting infiltrating macrophages by using λ-carrageenan type IV or a small molecule β2 integrin agonist leukadherin-1 (LA1) significantly suppressed cardiac and renal vasculopathy, alleviated chronic tissue injury and dysfunction (67, 68). Although increasing evidence suggest that macrophage-mediated vasculopathy is an integral part of the chronic allograft rejection process, the underlying mechanisms have not been well-defined. Distinct macrophage populations and functions in the process of allograft vasculopathy still remain to be further studied.

## Macrophages, Adaptive Immune System and Chronic Allograft Rejection

It has been widely recognized that adaptive immune response mediated by T and B lymphocytes, and donor-specific antibodies (DSA) are all involved in culminating chronic allograft rejection (69). In consideration of important roles of macrophages in the development of transplant rejection, questions concerning the link between macrophages and adaptive immune system need to be addressed. Despite the potential effects of CD4+ T cells in macrophage-mediated fibrotic rejection, which we have mentioned above, interestingly, recent arguments are challenging the traditional perspective on macrophage as innate immune cells. Chu et al. have advocated the concept that macrophage is potential to represent adaptive immune feature in allograft rejection, stating that CD4^+^T cells facilitate the acquisition of long-term specific memory of macrophages against skin allograft (70, 71). Moreover, another study demonstrates that macrophages can produce B-cell activating factor (BAFF), resulting in graft damage during antibody mediated rejection (72). Thus, in spite of the innate immune features of macrophages, mechanistic appreciation of their roles in connecting with adaptive immune system will be of interest in the future.

## Conclusion and Future Remark

Lots of efforts have been made to dissect the mechanisms of both innate and adaptive immune response so as to prevent chronic rejection and improve allograft survival (73). At present, a remarkable progress has been made in the study of macrophage-related immunoregulation and immunosuppression for the treatment of organ transplant rejection. Inhibiting infiltration or activation of monocytes/macrophages by mycophenolate mofetil (MMF) seems to be a promising therapeutic strategy (74). Indeed, depletion of monocytes/macrophages attenuate neutrophils-related tissue damage at the early stage of transplant (75–77), while it may also prevent tissue repair and induction of tolerance at late stage due to loss of reprogrammed macrophages that participate in reconstitution of homeostasis (17, 78). Moreover, chronic rejection mediated by M2-like macrophages is also an ineligible issue. Manipulating macrophage activities and polarization will be of significant challenge in against chronic allograft rejection.

## Author Contributions

HZ initially collected literatures and primarily drafted the article. ZL performed an intensive revision of the manuscript. WL contributed to the conceptual design and carried out critical revision and finalization of the manuscript. All authors contributed to the article and approved the submitted version.

## Conflict of Interest

The authors declare that the research was conducted in the absence of any commercial or financial relationships that could be construed as a potential conflict of interest.
